# Royal Medical and Chirurgical Society

**Published:** 1900-03-31

**Authors:** 


### ROYAL MEDICAL AND CHIRURGICAL SOCIETY.

At the meeting of this society on Tuesday two very interesting papers were read, one showing the possibility of the œsophagus becoming ruptured by the act of vomiting, and suggesting that such an accident may not be nearly as uncommon an event as is generally imagined; and the other illustrating on the one hand what good surgery can do in removing enormous tumours, and, on the other hand, showing how the most generally accepted rules as to prognosis may be falsified by the event in exceptional cases; for here we had an enormous tumour, which, according to its microscopical examination, was of a malignant character, which clinically had shown its malignancy by its rapidity of growth, and which, moreover, from its position, had to be "shelled out" without any of that "cutting clear of the affected area" which is now regarded as so important in dealing with malignant growths, and yet four years after the operation the patient remains in good health without any signs of recurrence.

The first case was related by Dr. Bowles and Mr. G. R. Turner. The patient, a female, aged 62, after taking overnight a pill of aloes and rhubarb, which acted freely, was sick on taking some milk. She vomited four or five times, and, still feeling ill, took a tumblerful of salt and water "to clear the stomach." The vomiting which this draught set up was followed by collapse of an alarming nature and epigastric pain. Some chlorodyne was at once administered by her maid, after swallowing which she complained of agonising pain. When first seen the symptoms were those of profound collapse; her respiration was gasping, and she was moaning with pain, which she referred to the epigastrium and the dorsal spine. There was retractation and some tenderness of the upper abdomen, with rigidity of the rectus. All vomiting had ceased since the sudden onset of the pain and collapse. Stimulants and laudanum given by the mouth immediately aggravated the pain. It was thought that some perforation of the stomach had possibly occurred, but the diagnosis was by no means clear. She rallied somewhat after the collapse, and under the influence of hypodermic morphine it became possible to examine her more thoroughly, although her gasping moans, extreme distress, and rapid respiration made auscultation difficult. At this time, some six hours after the attack, the symptoms were evidently more thoracic than abdominal, and it was decided that there was no indication for laparotomy. Under opiates she was kept quiet, but when roused the pain returned. Then there came emphysema of the neck, soon extending to the face, and she died about 22 hours after the attack.

The post-mortem showed that about one and a-half inches above the diaphragm there was a longitudinal rupture of the œsophagus five-eighths of an inch in length, with thin edges, but with no peeling of the mucous membrane. There was pneumo-thorax on the left side, with about six ounces of brownish fluid in the left pleural cavity and a small quantity also in the right cavity, the posterior mediastinum being infiltrated with similar fluid. The lungs were healthy—the left one collapsed. No tubercle, no adhesions, no rupture of visceral pleura, and no signs of other disease of any kind. A very careful review of the literature of the subject, with a table of collected cases, was appended to the paper, and the authors discussed the possibility of operative treatment.

We fully agree with much that was said in the discussion as to the possibility, and, in fact, the probability that this accident may occur more frequently than is generally supposed. It is clear that the collapse was in this case so profound that a very little more would have killed the patient right off, and it would be by no means difficult in such a case to make an erroneous diagnosis of death from angina brought on by the straining of the vomiting.

In regard to operation, of course, the œsophagus can be got at, although with difficulty; but, as was pointed out in the discussion, where the accident itself involves the pleura there need be no hesitation in going through this cavity, which would much facilitate matters.

The second paper was by Mr. Pearce Gould, who related a case in which he had removed a large sarcomatous tumour from the abdomen. When operated upon it was found to occupy the gastro-hepatic omentum, and the stomach was displaced into the pelvis. The tumour weighed 21 lb., and microscopic examination showed it to be a spindle-celled sarcoma with extensive hæmorrhages into its tissue. The patient recovered and remains well without a sign of recurrence—a period of four years and a half. The author drew attention to the following points: (1) To the extreme rarity of primary tumours in the gastro-hepatic omentum; he has found a record of only one other case. (2) To the means of diagnosing a tumour in this situation. (3) To the very great elongation and displacement of important structures which occurred without serious symptoms, and was recovered from without ill-effect. (4) To the long period of freedom from recurrence after removal of a sarcoma of apparently malignant character.

